# Assessment of Heavy Metal Concentrations in Pawpaw (Carica papaya Linn.) around Automobile Workshops in Port Harcourt Metropolis, Rivers State, Nigeria

**DOI:** 10.5696/2156-9614-7.14.48

**Published:** 2017-06-22

**Authors:** Olatunde Sunday Eludoyin, Onisoya Margaret Ogbe

**Affiliations:** Department of Geography and Environmental Management, University of Port Harcourt, Port Harcourt, Nigeria

**Keywords:** soil pollution, auto mechanic, heavy metals, bio-accumulation, human health

## Abstract

**Background.:**

Consumption of plants such as Carica papaya grown around automobile workshops is common in big cities in Nigeria. However, little is known about the heavy metals contamination of these consumables due to the influence of automobile emissions during maintenance activities.

**Objectives.:**

This study aimed to assess heavy metal concentrations in C. papaya and supporting soils around automobile workshops in Port Harcourt Metropolis, Rivers State, Nigeria.

**Methods.:**

Seven automobile workshops were used for the present study. First, 20 m × 20 m quadrats were laid out for soil and C. papaya tissue sampling. One composite soil sample was collected from the topsoil (0–15 cm depth) around each of the automobile workshops. Three C. papaya stands at least 30 cm apart around each workshop were used for the study and from these stands, tissues (root, stem, leaf, fruit) of C. papaya were collected. Standard laboratory techniques were used to determine the pH, electrical conductivity (EC) and heavy metals (lead (Pb), mercury (Hg), cadmium (Cd), copper (Cu), and zinc (Zn)) in the soil samples and C. papaya tissues. Pairwise t-test was used to determine significant differences (p<0.05) in the heavy metal concentrations in soil and C. papaya tissues between the sample and control sites, while correlation statistics were used to determine the relationship of heavy metal concentrations between soil and C. papaya tissues.

**Results.:**

C. papaya tissues and supporting soil had significantly higher levels of pH, EC and heavy metals in the sampled plots than the control plot. The heavy metal concentrations in C. papaya and soil occurred in the decreasing order of Pb>Cu>Hg>Zn>Cd. The fruit of C. papaya had the highest mean concentrations of Pb (51.4±14.1 mg/kg) and Zn (26.4±1.9 mg/kg), while the leaf had the highest mean concentration of Hg (32.0±2.3 mg/kg). The pH, Cu and Zn in the supporting soil were significantly correlated with the levels in the C. papaya tissues.

**Conclusion.:**

Bio-accumulation of heavy metals by C. papaya is evident around automobile workshops, and Pb, Hg, Cd concentrations were found to be above the permissible limits for human consumption according to World Health Organization (WHO) standards. Consumption of food materials grown around automobile workshops could pose health risks.

## Introduction

Automobile workshops are sources of auto wastes in cities and pose environmental hazards in developing countries. Auto wastes include used oil and fluids, dirty shop rags, waste from solvents used for cleaning parts, metallic particles from machinery wear, used batteries, asbestos from brake pads, oxidation products, and organic and inorganic chemicals used in oil additives and metals.^1^,^2^ The release of automobile wastes is responsible for the accumulation of heavy metals in soils and vegetation around the automobile workshops.^3^ Automobile workshops accumulate heavy metals concentrations in soil, creating environmental and health hazards.^4^ The soils in the tropics are capable of supporting complete food production and modifying carbon cycling, but soil pollution from automobile waste has devastated the soil.^5^ Automobiles in Nigeria mainly use petrol and the smoke releases metallic oxides, principally lead oxide and dust.^6^ Although use of leaded petrol has been reduced in some countries, an upsurge in automobile numbers has countered the effect of this reduction on vehicle-based lead(II) ion (Pb^2+^) production.

In urban soil, micro-nutrient (zinc (Zn), lead (Pb) and copper (Cu)) enhancement is due to automobiles and other factors.^7^ The effects of contaminants from automobile workshops have reached an alarming rate in Nigeria as heavy metal contamination has affected food at the trophic level as well as organisms.^8^ Soil pollution is a main cause of soil resource degradation.^9^ Heavy metals are absorbed by growing crops and transferred to other parts of the plant.^10^ The destroyed soil leads to low yields in crop production and low quality crops.^11^,^12^ Vegetables, tubers, flowers and trees such as pawpaw (Carica papaya) and other medicinal plants are often cultivated around these automobile workshops.

There have been a number of studies on heavy metals in soils and plants, including an assessment of heavy metal contents in roadside dust of Dhaka Metropolitan City, Bangladesh, a study of the effects of automobile fumes on the concentration of Pb in bread in Port Harcourt City, Nigeria, a study of the concentration of Pb in sweet orange in Port Harcourt, and an examination of the influence of automobile workshops along major roads of Port Harcourt City on soil properties.^3^,^7^,^13^,^14^ Other studies include an examination of the levels of total hydro-carbon in plants along the roads Port Harcourt City, the effects of heavy metals on topsoil at the vicinity of automobile mechanic villages in Owerri, Nigeria, and the level of pollution of the soil caused by automobile workshop waste.^15–20^ None of these studies have investigated the influence of automobile workshop wastes on C. papaya, despite the fact that it is a common plant growing naturally around mechanic workshops in Nigeria. C. papaya is a fruit commonly consumed all around the world, especially in the developing world. This is due in part to its reputation for having medicinal properties.^21^ This study examined the impact of automobile wastes on the tissues (root, leaf, bark and fruit) of C. papaya in Port Harcourt Metropolis, Nigeria.

**Abbreviations:**
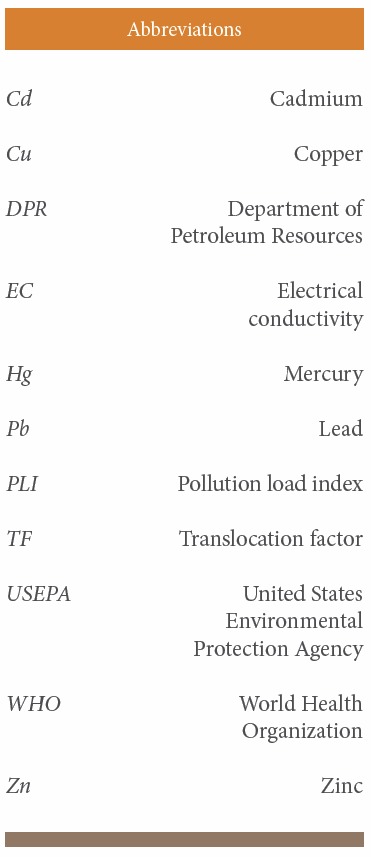


## Methods

### Study Site

**Figure 1:**
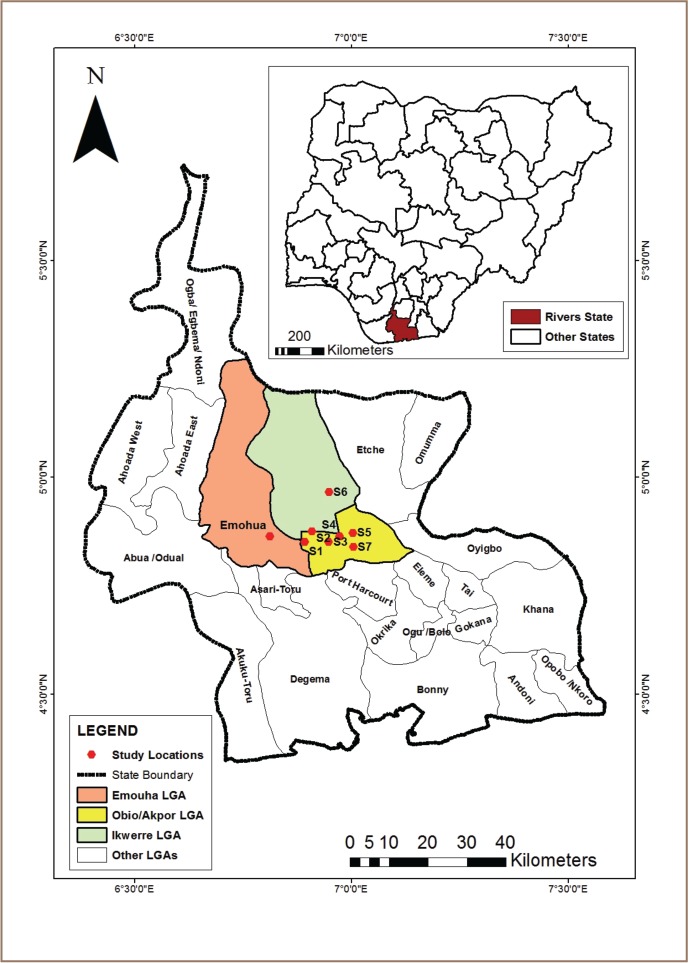
Rivers State, Showing the Study Locations Inset: Nigeria, Showing Location of Rivers State

The study was carried out around mechanic workshops in selected communities in Obio/Akpor, Ikwerre and Emohua Local Government Areas of Rivers State, Nigeria. The communities included Choba, Alakahia, Rumuosi, Rumualogu, Igwuruta and Emohua. An automobile mechanic workshop was selected in each of the communities based on the abundance of pawpaw in that environment and whether the automobile workshop had been in existence for at least 10 years. All study locations had the same soil type, climate and ecological zone (rainforest). The study area was located between latitudes 4°51′ 30″N and 4° 57′ 30″N and longitudes 6°50′ 00″E and 7°00′ 00″E *(Figure 1),* in the sub-equatorial region with a tropical climate. The mean annual temperature and relative humidity are 30°C and 85%, respectively. The rainfall is seasonal, variable and heavy, and mean annual rainfall is about 2,300 mm.^22^ The prevailing wind is basically south-westerly and north-easterly, with a wind speed between 5 and 17 m/s.^23^ The topography of the study area ranges between 16 m and 40 m above sea level.^22^ The vegetation type resembles that of a tropical rainforest. The vegetation is consistently nourished with high rainfall and high temperatures, providing favorable conditions for the growth of varieties of tall trees such as Swietenia macrophylla, Triplochiton scleroxylon, Terminalia superba, Elaeis guineensis and Raphia hookeri.^24^ The soils of the area can be categorized as freshwater brown and sandy loams.

### Research Design and Site Description

The study included experimental plots (automobile workshops) and a purposively selected control plot (natural forest). The control plot is about 1000 m away from any automobile shops or dumpsites of any sort. Seven automobile workshops were identified at least 200 m apart and with respect to their locations, these included S1 (Wilbros); S2 (UNIPORT); S3 (Choba); S4 (Alakahia); S5 (Rumuosi); S6 (Igwuruta); S7 (Rumualogu) and the control (Emohua). Study criteria for the sampled workshops were existence for a minimum of 10 years and an abundance of pawpaw around the workshop. C. papaya was selected for the study because of its abundance around all of the automobile workshops. In addition, C. papaya produces edible fruit, and the leaf, stem and roots are used to treat various illnesses. The soil around the automobile workshops is black in color as a result of petrol, diesel, engine oil and spray paints. In addition, there are piles of automobile waste products including containers for spray paints, thinner, petrol, diesel, and residue of carbide (CaC_2_) around the automobile workshops.

### Collection of Carica papaya Leaf, Stem, Root and Fruit and Soil Samples

A test plot measuring 20 m × 20 m was laid out around the pile of refuse in each automobile workshop and a control plot also measuring 20 m × 20 m was sited on the floor in the natural forest in Emuohua. In each quadrat, three mature C. papaya plants measuring 30 m were identified. Soil samples were collected from the topsoil (0–15 cm) around the identified C. papaya plants. Three soil samples were collected around each C. papaya stand with a soil auger and mixed in a bowl. A composite sample was taken from the C. papaya stand and put into a labeled polythene bag. Altogether, 21 soil samples were collected from both the test and control plots and taken to the laboratory for further analysis. C. papaya leaf, stem, root and fruit were collected from the same C. papaya stand identified for soil sample collection. From each C. papaya stand, a total of three leaf, stem (base, middle, top), root and fruit samples were collected. Papaya leaves, stems, roots and fruits collected from each stand were homogenized to make a composite sample of each of the three sites and labeled accordingly.^25^ The C. papaya tissue sample collection was performed using a stainless steel knife. All samples were well washed with distilled water to remove adsorbed soil particles and then air-dried.

### Laboratory Analysis of Carica papaya Tissues

Fruit samples were scraped to separate the seed from the fruit and thereafter only the fruit was crushed. Subsequently, the leaves, stems and roots were crushed. All tissues were oven-dried at 70°C for 24 hours. After cooling at ambient temperature, the dried tissues were milled into fine powder and sieved using a 2 mm mesh and thereafter kept in pre-cleaned screw-capped polyethylene containers for further analysis. The sample digests of the fruit, leaves, stems and roots were analyzed in three replicates for pH, electrical conductivity (EC), Pb, cadmium (Cd), Cu, mercury (Hg) and Zn. The heavy metals (Pb, Cd, Cu, Hg and Zn) in the C. papaya tissues were determined by atomic absorption spectrophotometry (Buck Scientific Model 210/211 VGP, US) using the Association of Official Agricultural Chemists (AOAC) standard.^26^ Then pH was determined using a water solution, while EC was determined using a conductivity meter.^27^ The elemental analyses were done in the agronomy laboratory of the University of Ibadan, Ibadan, Nigeria.

### Laboratory Analysis for Soil Samples

Soil pH was measured potentiometrically in 0.01 M calcium chloride solution using 1:2 soil solutions. Electrical conductivity was determined using a 1:2 ratio of soil sample to distilled water measured with a digital conductivity meter.^25^ Extracts used for determining heavy metals (trace elements) were obtained by leaching soil samples using 0.1 N ethylenediaminetetraacetic acid (EDTA) and 5 g of each sample was weighed into a clean, dry silica dish, covered and ignited in a furnace for 6 hours at 500°C until a grey white ash was obtained.^28^ The cover of the dish was opened to allow for escape of gases. To cool ash samples, 5 ml of 10% hydrochloric acid was added to enhance dissolution and 5 ml of 10% nitric acid was subsequently added and set in a water bath to dissolve completely. The solution was later relocated into a clean dry 50 ml standard volumetric flask and made to mark with distilled water.^29^ The concentrations of extractable trace metals including Cu, Pb, Cd, Hg and Zn were determined using atomic absorption spectrophotometry (Buck Scientific Model 210 VGP, USA).^30^ The blank reagent and standard reference soil materials were included in each sample batch to verify the accuracy and precision of the digestion procedure and for subsequent analyses. All samples were quantified in quadruplicate. The elemental analyses were performed in the agronomy laboratory of the University of Ibadan, Ibadan, Nigeria.

### Method of Data Analysis

Descriptive and inferential statistics were applied in the present study. The mean pH, EC, Pb, Cu, Cd, Hg and Zn in the tissues of C. papaya and soils were explained using descriptive statistics (mean and standard deviation). Pairwise t-test and Pearson's correlation statistics were applied for inferential statistics. Pairwise t-test was used to determine significant differences in pH, EC, Pb, Hg, Cu, Cd and Zn in the tissues of C. papaya and soil in the study area between the test and control sites. Pearson's correlation was used to determine the relationships in the heavy metals between the soil and C. papaya. The mean values of the heavy metals in soil were compared with the permissible levels set by the United States Environmental Protection Agency (USEPA) and Department of Petroleum Resources (DPR) in Nigeria, while those of C. papaya tissues were compared with the permissible levels of the World Health Organization (WHO). The pollution load index (PLI) was evaluated for each study site.^31^ The PLI was obtained as a contamination factor for each metal with respect to the natural background value in the soil was computed using Equations 1 and 2.^32^ The plant/soil metal concentration ratio and translocation factor were determined.^32^

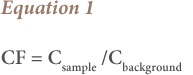


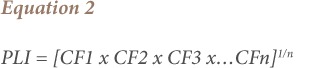
*Where,*

*CF*: *contamination factor,*
*n*: *number of metals = 5;*
*C_sample_*: *metal concentrate in polluted soils;*
*C_background_*: *mean natural background value of that metal.*

### Quality Assurance and Quality Control

#### Field Procedures

All field procedures were in accordance with general and standard Quality Assurance and Quality Control requirements of the USEPA.^33^ Contamination of samples was avoided by using clean and sterile sampling containers. Separate samples were used for analyzing parameters requiring different treatment or preservation before analysis. A composite sampling technique was adopted for soil.^34^ Control samples were collected at appropriate points remote from focus areas (about 1000 m away from the nearest automobile workshop). Samples were appropriately preserved and labeled. Proper chain of custody was applied.

#### Laboratory Procedures

Analyses were carried out within the holding time of respective parameters. Only functional and calibrated equipment was used for sample analysis. Only trained and experienced staff were involved in the analytical work.

## Results

### Effects of automobile workshops on heavy metals uptake in Carica papaya tissues

**Figure 2:**
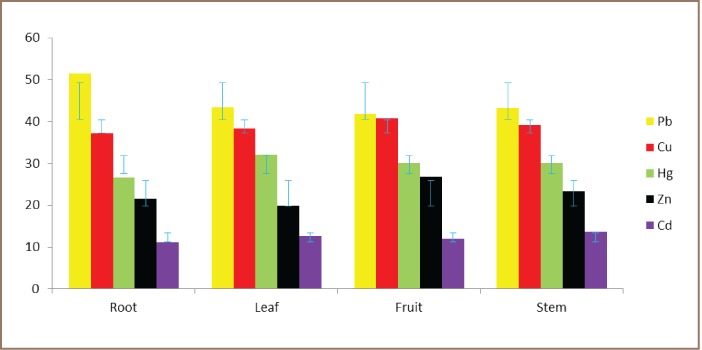
Trend of heavy metals concentrations in Carica papaya tissues

Findings revealed that pH ranged between 5.0 and 6.1 in the root of C. papaya with mean pH of 5.67 around automobile workshops, while in the control site, pH ranged between 7.8 and 8.3 with a mean pH of 8.09. Similarly, the mean pH in the leaf, fruit and stem were lower than that of the control plot, suggesting that the pH level in the C. papaya tissues around the automobile workshops was acidic and alkaline in the control plot. Differences between the control and test plots are shown in Table 1. The mean EC in the root, leaf, fruit and stem at the polluted site was 1386.1 μS/cm, 1580.2 μS/cm, 1451.4 μS/cm and 1401.1 μS/cm, respectively, while the EC at the control site was 357.7 μS/cm, 419.4 μS/cm, 439.7 μS/cm and 390.3 μS/cm in the root, leaf, fruit and stem, respectively (Table 1). The root had the highest mean Pb level (51.4 mg/kg), while the lowest level was found in the fruit, with a mean Pb level of 41.8 mg/kg at the test sites *(Table 1).* In the control plot, the root of C. papaya had the highest mean Pb level (18.5 mg/kg) and the lowest level was found in the fruit, with a mean Pb level of 14.8 mg/kg at the control plot *(Table 1).* The analysis revealed that Hg concentration varied slightly among the tissues of C. papaya with the highest concentration in the leaf (32.0 mg/kg) around the automobile workshops *(Table 1).* Furthermore, mean Cd in the root was 11.1 mg/kg, with levels ranging from 2.3–16.5 mg/kg, while the mean Cd in the leaf, fruit and stem was 12.7 mg/kg, 11.9 mg/kg and 13.6 mg/kg, respectively, around the auto mechanic workshops *(Table 1).* At the control plot, the highest mean Cd was found in the root (3.4 mg/kg) *(Table 1).* The mean Cu in the root was highest in the fruit with a mean value of 40.8 mg/kg around the mechanic workshop, while the mean Cu was highest in the root, with a mean value of 6.5 mg/kg in the control plot. The concentration of Zn in the root ranged between 2.6 mg/kg and 63.0 mg/kg, with a mean Zn of 21.5 mg/kg at the test sites. Similarly, the concentration of Zn ranged from 19.8 mg/kg in leaf to 26.4 mg/kg in fruit at the test sites. The concentration of Zn was highest in the root (2.9 mg/kg) in the control plot. All investigated heavy metals were significantly higher around automobile workshops compared to the control plot *(Table 1).* Generally, the mean concentrations of heavy metal contents in the tissues of C. papaya around the automobile workshops occurred in the decreasing order of Pb>Cu>Hg>Zn>Cd *(Figure 2).* Comparing the level of concentrations of heavy metals with the WHO permissible levels, all investigated heavy metals were higher than the permissible level, indicating that the plants were polluted *(Table 1).*

**Table 1:** pH, EC and Heavy Metals in Carica papaya

**Parameter**	**AUTOMOBILE WORKSHOP PLOTS**				**CONTROL PLOT**			**t value (p value)**	**WHO Maximum Limit**
	**Tissue**	**Minimum**	**Maximum**	**Mean±SD**	**Minimum**	**Maximum**	**Mean±SD**		
**pH**	Root	5.00	6.10	5.67±0.43	7.80	8.30	8.09±0.18	22.756 (0.005)^*^	-
	Leaf	5.10	6.00	5.56±0.40	7.90	8.70	8.16±0.26		
	Fruit	5.00	6.20	5.67±0.46	7.80	8.40	8.19±0.25		
	Stem	5.20	6.30	5.81±0.44	7.80	8.60	8.17±0.25		
**EC (μS/cm)**	Root	868.0	2120.0	1386.1±389.0	43.0	570.0	357.7±106.7	11.932 (0.001)^*^	-
	Leaf	724.0	2123.0	1580.2±466.2	310.0	504.0	419.4±64.4		
	Fruit	893.0	2111.0	1451.4±428.8	275.0	610.0	439.7±109.7		
	Stem	853.0	2146.0	1401.1±433.5	280.0	506.0	390.3±71.5		
**Pb (mg/kg)**	Root	37.50	75.10	51.4±14.1	10.80	26.00	18.5±5.3	10.243 (0.001)^*^	0.3^**^
	Leaf	21.30	80.30	43.4±19.3	8.20	28.50	17.6±7.1		
	Fruit	18.40	65.20	41.8±16.6	7.30	20.40	14.8±5.8		
	Stem	25.30	70.50	43.2±15.4	8.60	19.60	14.9±4.4		
**Hg (mg/kg)**	Root	3.9	36.7	26.7±11.4	6.5	19.5	12.6±5.1	11.760 (0.001)^*^	0.05–0.5^****^
	Leaf	28.3	34.8	32.0±2.3	6.7	17.2	11.2±3.6		
	Fruit	19.2	38.6	30.2±5.9	5.1	13.7	9.7±2.9		
	Stem	18.6	41.0	30.2±6.7	6.5	15.1	9.7±3.1		
**Cd (mg/kg)**	Root	2.3	16.5	11.1±4.9	1.5	6.3	3.4±0.6	12.332 (0.001)^*^	0.2^**^
	Leaf	9.0	17.1	12.7±2.5	1.3	4.8	2.8±0.4		
	Fruit	5.5	15.0	11.9±3.6	9.0	4.0	2.8±0.3		
	Stem	8.0	18.0	13.6±3.1	1.0	3.2	2.0±0.2		
**Cu (mg/kg)**	Root	18.7	73.2	37.1±2.1	1.6	6.5	2.1±1.6	8.329 (0.001)^*^	73^***^
	Leaf	19.0	70.4	38.3±2.2	2.0	5.1	3.5±1.4		
	Fruit	17.5	79.1	40.8±2.3	1.8	5.5	3.0±1.3		
	Stem	15.6	88.0	39.2±2.8	1.5	4.9	2.9±1.2		
**Zn (mg/kg)**	Root	2.6	63.0	21.5±2.0	1.7	6.7	2.9±2.5	6.143 (0.001)^*^	100^***^
	Leaf	2.3	57.0	19.8±1.8	1.6	3.4	2.2±0.6		
	Fruit	3.1	49.0	26.4±1.9	1.40	3.1	2.2±0.5		
	Stem	1.8	44.2	23.3±1.4	1.7	4.0	2.3±0.8		

**Abbreviations:** ^*^t, significant at p<0.05; n, 7; SD, Standard Deviation.

**Sources:** WHO Maximum Limit of Heavy Metals in C. papaya^****^;^35^^**^;^36^^***^;^37–39^

### Effects of Automobile Workshops on pH, EC and Heavy Metals in Soil

The pH level at the test site was significantly more acidic than at the control plot (t=6.074, p<0.05). Similarly, the mean EC was significantly higher in soils at the test site than the control site (t=4.275, p<0.05). The concentration of Pb in the polluted soil ranged between 56.9 mg/kg and 93.4 mg/kg with a mean Pb of 73.6 mg/kg, whereas in the control site, Pb concentration ranged between 15.8 mg/kg and 36.7 mg/kg with mean Pb of 21.9 mg/kg *(Table 2).* Among the investigated heavy metals, Pb was the highest in both polluted and control soil. Furthermore, the mean Hg, Cd, Cu and Zn concentrations in the polluted soil were 38.4 mg/kg, 20.3 mg/kg, 56.1 mg/kg and 47.8 mg/kg, respectively *(Table 2).* Compared with control samples, the concentrations of Hg, Cd, Cu and Zn were significantly higher in the test samples (Table 2). The concentrations of Hg, Cd and Cu around the automobile workshops were higher than the permissible limit of the DPR, while the concentrations of Pb, Cd, Cu and Zn from the test and control plots were lower than the permissible limits of the USEPA *(Table 2).* Similar to the tissue of C. papaya, the mean concentrations of heavy metal contents in soils around the mechanic workshops occurred in the decreasing order of Pb>Cu>Hg>Zn>Cd.

**Table 2:** pH, EC and Heavy Metals Concentrations in Soil

**Soil Parameters**	**AUTOMOBILE WORKSHOP PLOTS**			**CONTROL PLOT**			**t value (p value)**	USEPA^**^	DPR^***^
	**Minimum**	**Maximum**	**Mean±SD**	**Minimum**	**Maximum**	**Mean±SD**			
**pH (H_2_O)**	3.7	5.4	4.7±0.7	6.4	6.9	6.6±0.2	6.074 (0.001)^*^	-	-
**EC (us/cm)**	1050.0	5240.0	1662.3±76.4	360.0	708.0	417.0±13.5	4.275 (0.005)^*^	-	-
**Pb (mg/kg)**	56.9	93.4	73.6±12.7	15.8	36.7	21.9±7.2	13.057 (0.000)^*^	300	85
**Hg (mg/kg)**	29.6	53.1	38.4±9.8	5.9	36.2	17.1±1.2	5.409 (0.002)^*^	-	0.3
**Cd (mg/kg)**	9.2	44.1	20.3±1.2	1.1	10.4	4.7±0.3	4.699 (0.003)^*^	70	0.8
**Cu (mg/kg)**	20.1	114.0	56.1±3.7	2.8	9.4	5.4±0.2	3.745 (0.01)^*^	250	36
**Zn (mg/kg)**	2.5	104.0	47.8±4.1	2.5	5.0	3.2±0.8	2.910 (0.027)^*^	300	140

**Abbreviations:** ^*^t, significant at p<0.05; n, 7; SD, Standard Deviation.

**Sources:** USEPA and DPR Maximum Allowable Limits of Heavy Metal in Soil ^**^;^40^^***^;^41^,^42^

### Contamination Factor and Pollution Loading Index of Heavy Metals in Soil

Contamination factor and pollution load index were used to assess heavy metal contaminations in soil located around the automobile workshops *(Table 3).* The contamination factors for the five metals in different locations were high, ranging from 0.8 to 32.5 in Zn. In all the locations, Zn had the highest contamination factor, except in S4, while Hg was the lowest, except in S4, S5 and S6. The contamination factors of all the heavy metals indicate severe to very severe contamination *(Table 4).* The PLIs of all the study locations were greater than unity and the majority indicated moderate pollution levels, except locations S5 and S6 (severe pollution levels) and S7 (very severe pollution) *(Table 3).*

**Table 3:** Contamination Factors of Heavy Metals in Soils Around Mechanic Workshops

**Heavy Metals**	**S1 Wilbros**	**S2 UNIPORT**	**S3 Choba**	**S4 Alakahia**	**S5 Rumuosi**	**S6 Igwuruta**	**S7 Rumualogu**	**Control Emohua**
**Pb**	2.9	3.2	3.9	4.3	2.6	3.1	3.6	21.9±7.2
**Hg**	1.7	1.7	2.3	1.8	2.9	3.1	2.1	17.1±1.2
**Cd**	2.0	2.8	3.7	2.5	5.7	4.2	9.4	4.7±0.3
**Cu**	5.1	7.1	3.7	5.2	21.1	12.6	17.9	5.4±0.2
**Zn**	9.2	8.6	2.7	0.8	30.0	20.9	32.5	3.2±0.8
**PLI**	**3.4**	**3.9**	**3.2**	**2.4**	**7.6**	**6.4**	**8.3**	

**Table 4:** Intervals of Contamination/Pollution Index

**Class**	**Contamination Factor Index**
<1	Very slight contamination
0.10–0.25	Slight contamination
0.26–0.5	Moderate contamination
0.51–0.75	Severe contamination
0.76–1.00	Very severe contamination
1.10–2.0	Slight pollution
2.1–4.0	Moderate pollution
4.1–9.0	Severe pollution
9.1–16.0	Very severe pollution
>16.0	Excessive pollution

**Source:** Lacatusu, 1998 43

### Plant/Soil Metal Concentration Ratio

Analysis of the plant/soil metal concentration ratio showed that Hg had the highest plant/soil metal concentration factor around automobile workshops (0.776), although its bioavailability in C. papaya (38.4 mg/kg) was lower than Pb (73.6 mg/kg), Cu (56.1 mg/kg) and Zn (47.8 mg/kg). The plant/soil metal concentration ratio trend around mechanic workshops was in the order of Hg (0.776) > Cu (0.693) > Pb (0.611) > Cd (0.605) > Zn (0.476). Lead and Zn had higher plant/soil metal concentrations, 0.753 and 0.750, respectively, at the control site.

### Translocation Factor

Translocation factor, the ratio of the concentration of metal in the aerial portion of the plant to the total concentration in the soil sample, is presented in Table 5. The analysis reveals that Hg, Cd, Zn and Cu had TFs of 3.46, 3.44, 3.19 and 2.49, respectively, while the highest TF in the control site was found for Cu (4.47). The TFs in the study area were generally very high, as they were greater than 1.

**Table 5:** Translocation Factor

**Heavy Metals**	**AUTOMOBILE WORKSHOPS (TEST PLOTS)**			**CONTROL PLOT**		
	**Aerial Portion**	**Root**	**TF**	**Aerial Portion**	**Root**	**TF**
**Pb**	128.4	51.4	2.49	47.3	18.5	2.56
**Hg**	92.4	26.7	3.46	30.6	12.6	2.43
**Cd**	38.2	11.1	3.44	7.6	3.4	2.24
**Cu**	118.3	37.1	3.19	9.4	2.1	4.47
**Zn**	69.5	21.5	3.23	6.7	2.9	2.31

### Relationships between pH, EC and Heavy Metals between Carica papaya Tissues and Soil

The correlation analysis revealed in Table 6 shows that pH in soil was positively correlated with pH in the root (r=0.785, p<0.05); leaf (r=0.761, p<0.05); fruit (r=0.800, p<0.05) and stem (r=0.809, p<0.05) of C. papaya. Furthermore, EC in soil was positively correlated with EC in the root (r=0.937, p<0.05) and stem (r=0.786, p<0.05) of C. papaya. However, although Pb and Hg in soil were positively correlated with Pb and Hg in C. papaya tissues, none of these associations were significant. Meanwhile, the correlation between Cd in soil and Cd in root was negative and moderate (r=−0.536, p>0.05). Cu in soil was positively correlated with Cu in the root (r=0.964, p<0.05), leaf (r=0.893, p<0.05), fruit (r=0.893, p<0.05) and stem (r=0.893, p<0.05) of C. papaya. Similarly, the analysis showed that Zn in soil was positively correlated with Zn in leaf (r=0.893, p<0.05), fruit (r=0.964, p<0.05) and stem (r=0.929, p<0.05) of C. papaya. However, negative correlations existed between Zn in soil and Pb in root (r=−0.857, p<0.05), leaf (r=−0.821, p<0.05), fruit (r=−0.857, p<0.05) and stem (r=−0.821, p<0.05). The pH in soil was significantly correlated with Pb in root, leaf, fruit and stem; Cu in root, fruit and stem; and Zn in root, leaf, fruit and stem.

**Table 6:** Relationship in pH, EC and Heavy Metals between Carica papaya Tissues and Soil

	**pH Soil**	**EC Soil**	**Pb Soil**	**Hg Soil**	**Cd Soil**	**Cu Soil**	**Zn Soil**
**pH Root**	0.785^*^	−0.111	0.185	−0.673	−0.778^*^	−0.630	−0.778^*^
**pH Leaf**	0.761^*^	−0.127	0.436	−0.670	−0.800^*^	−0.655	−0.855^*^
**pH Fruit**	0.800^*^	−0.144	0.198	−0.673	−0.829^*^	−0.793^*^	−0.667
**pH Stem**	0.809^*^	−0.054	0.252	−0.573	−0.739	−0.847^*^	−0.685
**EC Root**	0.336	0.937^*^	0.126	0.555	−0.198	−0.378	−0.432
**EC Leaf**	0.306	0.571	−0.036	0.054	−0.607	−0.500	−0.429
**EC Fruit**	0.234	0.679	−0.036	0.198	−0.393	−0.571	−0.393
**EC Stem**	0.414	0.786^*^	0.250	0.306	−0.393	−0.571	−0.571
**Pb Root**	0.883^*^	0.071	0.429	−0.378	−0.571	−0.429	−0.857^*^
**Pb Leaf**	0.901^*^	0.002	0.571	−0.360	−0.464	−0.500	−0.821^*^
**Pb Fruit**	0.901^*^	−0.214	0.536	−0.649	−0.679	−0.571	−0.857^*^
**Pb Stem**	0.883^*^	−0.071	0.250	−0.667	−0.929^*^	−0.643	−0.821^*^
**Hg Root**	0.282	0.667	−0.252	0.464	−0.090	0.162	−0.180
**Hg Leaf**	−0.342	0.321	−0.821^*^	0.270	0.036	0.071	0.464
**Hg Fruit**	−0.721	0.071	−0.321	0.396	0.571	0.536	0.536
**Hg Stem**	−0.432	0.429	−0.393	0.396	0.107	−0.143	0.357
**Cd Root**	0.775^*^	0.536	0.607	0.018	−0.536	−0.643	−0.893^*^
**Cd Leaf**	0.126	0.143	0.750	0.162	0.015	−0.143	−0.321
**Cd Fruit**	0.018	−0.214	0.857^*^	0.018	0.214	−0.143	−0.143
**Cd Stem**	0.073	−0.342	0.505	−0.309	0.018	−0.505	−0.144
**Cu Root**	−0.829^*^	−0.286	−0.321	0.360	0.786^*^	0.964^*^	0.821^*^
**Cu Leaf**	−0.685	−0.357	−0.143	0.288	0.821^*^	0.893^*^	0.679
**Cu Fruit**	−0.883^*^	−0.107	−0.679	0.450	0.786^*^	0.893^*^	0.929^*^
**Cu Stem**	−0.883^*^	−0.107	−0.679	0.450	0.786^*^	0.893^*^	0.929^*^
**Zn Root**	−0.775^*^	−0.286	−0.214	0.054	0.357	0.214	0.643
**Zn Leaf**	−0.937^*^	−0.214	−0.500	0.252	0.536	0.536	0.893^*^
**Zn Fruit**	−0.991^*^	−0.143	−0.536	0.414	0.714	0.714	0.964^*^
**Zn Stem**	−0.991^*^	−0.179	−0.464	0.414	0.786^*^	0.786^*^	0.929^*^

^*^Correlation is significant at the 0.05 level

## Discussion

The pH in the tissues of C. papaya and soil around the automobile workshops was acidic in nature. However, previous studies have reported that the soil around the automobile workshops was relatively alkaline.^44–46^ The soil pH around the automobile workshop in Ikare Akoko ranged from 6.3–7.1.^46^ It was also reported that the soil pH of dumpsites in Kaduna was alkaline (7.85–8.60).^44^ However, it has been reported that soil pH around automobile workshops in the Gboko area of Benue State was weakly acidic.^47^ Soil acidity enhances the washing away of valuable chemicals such as Mg from soil, releasing Hg, Pb, and Cd into streams and rivers.^48^ Therefore, pH plays a significant role in solute concentration and in sorption and desorption of contaminants in soil.^46^ The EC around the automobile workshop was significantly higher in both C. papaya and soil around the automobile workshops compared to the control plot. The high EC indicates that high ions (salt) are present.^49^ This high electrical conductivity in the soil around the automobile workshop may be attributed to low pH.^50^

The results of this study indicate that the heavy metals under investigation are greatly contaminating the soil around the automobile workshops. The findings that the contamination factor, translocation factor and pollution load index are above the normal limits suggest the need for metals detoxification and examination of the disposal culture in automobile workshops.^16^,^45^,^47^ The PLI findings are in agreement with findings of Obasi *et al.* 2013, Ololade (2014), and Ogunmodede *et al.* 2016. 32,45,46 The translocation factor of Cd, Pb and Zn were greater than unity, similar to findings of previous studies.^32^,^51^ The higher concentrations of Cd, Pb and Zn in some plant species may be due to foliar absorption.^32^ Interestingly, the high transfer factor in C. papaya may help to easily translocate heavy metals from the roots to shoots. Plant species with high translocation factor values are considered suitable for phyto-extraction, as they generally translocate heavy metals to easily harvestable parts (shoots).^45^,^52^ Findings have revealed that Hg had the highest translocation factor of 3.46 and plant/soil metal ratio of 0.776, despite lower bioavailability compared to Pb and Cu. Findings also revealed that the Hg concentration in the study area was significantly higher than the permissible level of 0.3 mg/kg in soil set by the DPR and 0.05 in plants as set by the WHO. Soil contaminated by Hg has the potential to enter the food chain through plants and livestock and lead to cardiovascular, renal, neurological and reproductive health disorders.^53^

The concentration of Cd was the lowest among the heavy metals investigated in both C. papaya and soil. The concentration was significantly higher at the automobile workshops compared to the control site and the permissible level of 0.8 mg/kg in soil as set by the DPR and 0.2 mg/kg in plants as set by the WHO. The high Cd levels in the soil samples of automobile workshop sites can be attributed to motor vehicle repair activities such as painting, soldering, brake fluid, engine oil, corrosion of metal, batteries and metal parts such as radiators, and indiscriminate dumping of waste products also introduces Cd into these sites.^47^ The general low concentration of Cd in the present study may be linked to low pH (strongly acidic), which generally increases the mobility of heavy metals.^16^,^54^ In addition, a large portion of soil Cd has been found to be soluble in pore water at a depth of 30 cm in contaminated urban woodland soil.^55^ However, Cd is more mobile and percolates faster than other heavy metals, especially Hg and Pb.^3^

The significantly high concentrations of heavy metals in C. papaya show the tendency of C. papaya to absorb and retain heavy metals in its tissues, especially around automobile workshop sites. C. papaya had the highest levels of all heavy metals compared to what was obtainable in A. hybridus, T. triangulare, L. aegyptiaca and I. batatas in dumpsites located in Okigwe, Nigeria.^45^ Thus, the level of metal uptake depends primarily on the plant species, its inherent controls and soil quality.^45^,^56^

The concentration of Pb was observed to be the highest in all of the tissues of C. papaya around the automobile workshops and significantly higher than the control site and the permissible levels of 0.3 mg/kg as specified by the WHO. The bioaccumulation of Pb was higher in the underground organs (rhizomes and roots) of Hieracium pilosella.^57^ The higher Pb concentration in C. papaya tissues and soils around automobile workshops is in agreement with findings of previous studies.^3^,^47^,^58–61^ The degree of contamination of Pb was found to be highest in soils around an automobile workshop in Benue State, Nigeria.^47^ In addition, Pb was significantly high in the peels and fruits of C. papaya in Sii and Zaakpon Communities of Ogoniland, Rivers State, Nigeria and the concentrations were above the permissible limit of 0.3 mg/kg.^61^ The higher level of Pb might be due to traffic volume, automobile workshops and the immobilization of Pb in the topsoil.^3^,^62^ The presence of Pb is aggravated when Pb is used as an additive in automobile exhaust emission and paints.^61–65^ The occurrence of Pb in C. papaya and soil can be considered to be an important pathway in the food chain, especially in children, as a high concentration of Pb could have toxic effects on plants and humans.^66^ Pb is known to be toxic even at low concentrations, especially in young children.^67^ It has been reported that automobile workshops may be used as playgrounds or be located near residential areas where children play about freely and it is possible for children to ingest Pb through hand to mouth behavior, especially during the developmental years from ages 0–2.^47^ Thus, ingestion is considered to be the most significant pathway for Pb, which could lead to sensory disturbances, kidney disorders and brain damage.^67^

The concentration of Cu was significantly higher in the soil and C. papaya tissues around the automobile workshops compared to the control plot and the permissible level of 36 mg/kg in soil as specified by the DPR. The high concentration of Cu may be dangerous as it damages the liver and kidney when it gets into the human body through the food chain. The presence of Cu may be attributed to automobile workshops, traffic densities and the use of auto paints.^62^,^68^

Findings also showed that heavy metals in soil were positively correlated with heavy metals in the tissues of C. papaya, except for Cd in root. This suggests that the absorption of heavy metals in the tissues of C. papaya was greatly controlled by the content of heavy metals in the soil solution and bioavailability of metals in the supporting soil, and as result, C. papaya can be potentially used in the bio-monitoring of environmental contamination of heavy metals.^25^,^57^ However, pH in soil significantly influenced Pb levels in all the tissues, Cu in the root, fruit and stem; and Zn in all the C. papaya tissues investigated. Zinc in soil was negatively and significantly correlated with Pb in root, leaf, fruit and stem. The high negative correlation between Zn in soil and Pb in tissues gives a strong indication that they have different sources of contamination in the environment.^69^,^70^

## Conclusion

Bio-absorption of heavy metals (Pb, Cu, Hg, Cd, Zn) by the tissues of C. papaya is evident around mechanic workshops in Port Harcourt Metropolis, and their uptake is directly linked to the supporting soil. The accumulation of heavy metals in the root, leaf, fruit and stem of C. papaya and soils around mechanic workshops was significantly higher than in the control plot. The heavy metal concentrations in the tissues of C. papaya and soil around mechanic workshops occurred in the decreasing order of Pb>Cu>Hg>Zn>Cd. Mercury had the highest plant/soil metal concentration ratio and translocation factor despite its lower bioavailability compared to Pb and Cu. Positive and significant correlations existed between the heavy metals in the tissues of C. papaya, except for Cd in root and heavy metal contents in soil. We strongly recommend that the consumption of the leaf, stem, root and fruit of C. papaya grown around automobile workshops be discouraged. Indiscriminate disposal of automobile waste should be properly examined by the government so that this practice can be reduced or completely eradicated. Finally, since C. papaya has the tendency and potential to absorb heavy metals, it can be grown in surplus around automobile workshops for phyto-extraction of heavy metals and further studies on the level of accumulation of heavy metals in the human body, especially among auto-mechanic technicians and residents living around the automobile workshops are needed.

## References

[1] European Environment Agency (EEA). Progress in management of contaminated sites (CSI 015). Europe Environmental Assessment Agency, Kongan, 6DK-1050, Denmark [Internet]. 2007 7 [modified 2015; cited 2017 Jun 11]. Available from: http://www.eea.europa.eu/data-and-maps/indicators/progress-in-management-of-contaminated-sites/progress-in-management-of-contaminated-1

[2] Olayiwola OA. Levels of Pb, Fe, Cd and Co in soils of automobile workshop in Osun State, Nigeria. J. Appl. Sci. Environ. Manage. [Internet]. 2011 6 [cited 2017 Jun 11]; 15 2: 279– 282 Available from: http://file.scirp.org/pdf/JEP_2014082613335296.pdf

[3] Utang PB, Eludoyin OS, Ijekeye CL. Impacts of automobile workshops on heavy metals concentrations of urban soils in Obio/Akpor LGA, Rivers State, Nigeria. Afr J Agric Res [Internet]. 2013 7 11 [cited 2016 Dec 26]; 8 26: 3476– 82. Available from: http://www.academicjournals.org/journal/AJAR/article-full-text-pdf/8C2CBC036504

[4] Najib NW, Mohammed SA, Ismail SH, Ahmad WA. Assessment of heavy metal in soil due to human activities in Kangar, Perlis, Malaysia. Int J Civil Environ Eng [Internet]. 2012 12 [cited 2016 Dec 26]; 12 6: 28– 33. Available from: http://www.ijens.org/Vol_12_I_06/129306-2525-IJCEE-IJENS.pdf

[5] Sanchez PA. Soil fertility and hunger in Africa. Science [Internet]. 2002 3 15 [cited 2017 Jun 5]; 295 5562: 2019– 20. Available from: http://science.sciencemag.org/content/295/5562/2019.long Subscription required to view. 10.1126/science.1065256 11896257

[6] Nawazish S, Hussain M, Ashraf M, Ashraf MY, Jamil A. Effect of automobile related metal pollution (Pb^2+^ & Cd^2+^) on some physiological attributes of wild plants. Int J Agric Biol [Internet]. 2012 [cited 2017 Jun 5]; 14 6: 953– 8. Available from: https://www.researchgate.net/publication/285944857_Effect_of_Automobile_Related_Metal_Pollution_Pb2 _Cd2_on_some_Physiological_Attributes_of_Wild_Plants

[7] Rakib MA, Ali M, Akter MS, Bhuiyan MA. Assessment of heavy metal (Pb, Zn, Cr and Cu) content in roadside dust of Dhaka metropolitan City, Bangladesh. Int Res J Environ Sci [Internet]. 2014 1 [cited 2017 Jun 5]; 3 1: 1– 5. Available from: http://www.isca.in/IJENS/Archive/v3/i1/1.ISCAIRJEvS-2013-246.pdf

[8] Campbell LM, Norstrom RJ, Hobson KA, Muir DC, Backus S, Fisk AT. Mercury and other trace elements in a pelagic Arctic marine food web (Northwater Polynya, Baffin Bay). Sci Total Environ [Internet]. 2005 12 1 [cited 2017 Jun 5]; 351–352: 247– 63. Available from: http://www.sciencedirect.com/science/article/pii/S0048969705004213 Subscription required to view. 10.1016/j.scitotenv.2005.02.043 16061271

[9] Mbagwu JS. Aggregate stability and soil degradation in the tropics [Internet]. Lecture given at the College on Soil Physics; 2003 Mar 3–21; Trieste Italy. Nsukka, Nigeria: University of Nigeria; [date unknown; cited 2017 Jun 5]. p 247– 52. Available from: http://users.ictp.trieste.it/~pub_off/lectures/lns018/22Mbagwu1.pdf

[10] Adewole MB. Phytoremediation of contaminated soils with some heavy metal using Helianthus annuus L. and Tithonia diversifolia (Hemsl) as influenced by fertilizer applications [Ph.D. thesis]. [Ibadan, Nigeria]: University of Ibadan; 2006 149 p.

[11] Rainbow RW. Integration of no-till and precision agriculture technologies and future challenges to conservation agriculture in Australia. : Goddard T, Zoebisch MA, Gan Y, Ellis W, Watson A, Sombatpanit S, No-till farming systems. Special publication No. 3. Beijing, China: World Association of Soil and Water Conservation; 2007 p 223– 46.

[12] Adeoye GO, Sridhar MK, Adeoluwa OO, Akinsoji NA. Evaluation of naturally decomposed solid wastes from municipal dumpsites for their manurial value in Southwest Nigeria. J Sustain Agric [Internet]. 2005 [cited 2017 Jun 5]; 26 4: 142– 52. Available from: http://www.tandfonline.com/doi/abs/10.1300/J064v26n04_09?journalCode=wjsa20 Subscription required to view.

[13] Weli VE, Iwowari FA The impact of automobile exhaust fumes on concentration levels of lead on bread in Port Harcourt City, Nigeria. Int J Environ Pollut Res [Internet]. 2014 9 [cited 2017 Jun 5]; 2 3: 57– 72. Available from: http://www.eajournals.org/wp-content/uploads/The-Impact-of-Automobile-Exhaust-Fumes-on-Concentration-Levels-of-Lead-on-Bread-in-Port-Harcourt-City-Nigeria.pdf

[14] Odimegwu VC. Spatial analysis of lead (Pb) concentration in sweet orange (Citrus sinensis) among varying land use in Port Harcourt City and environs [dissertation]. [Port Harcourt City, Nigeria]: University of Port Harcourt; 2014 78 p.

[15] Ideriah TJ, Emerhi EA, Abere SA, Trinya W. Levels of total hydrocarbon contents in plants along selected roadsides in Port Harcourt Metropolis, Nigeria. J Agric Biol Sci [Internet]. 2011 6 [cited 2017 Jun 5]; 2 3: 65– 70. Available from: https://www.researchgate.net/profile/Simbi_Wellington_Werisuo/publication/276144822_TJK_Ideria_EA_Emerhi _SA_Abere_and_W_Trinya2011Levels_of_Total_Hydrocarbon_Contents_in_Plants_along_Selected_Roadsides_in_Port_Harcourt_ Metropolis_Nigeria/links/5551221d08ae93634ec9f90c.pdf?origin=publication_detail

[16] Okoro AC, Chukwuma GO, Chukwuma EC, Nwachukwu PC, Ezeh KA. Investigating the effects of selected heavy metals on topsoil at the vicinities of two automobile mechanic villages, Owerri Municipal, Nigeria. Int J Eng Sci [Internet]. 2013 [cited 2017 Jun 5]; 2 11: 19– 26. Available from: http://www.theijes.com/papers/v2-i11/Part.4/D021104019026.pdf

[17] Idugboe SO, Tawari-Fufeyin P, Midonu AA. Soil pollution in two auto-mechanic villages in Benin City, Nigeria. IOSR J Environ Sci Toxicol Food Technol [Internet]. 2014 2 [cited 2017 Jun 5]; 8 1: 9– 14. Available from: http://www.iosrjournals.org/iosrjestft/papers/vol8-issue1/Version-5/B08150914.pdf

[18] Iwegbue CM. Metal fractionation in soil profiles at automobile mechanic waste dumps. Waste Manag Res [Internet]. 2007 12 1 [cited 2017 Jun 5]; 25 6: 585– 93. Available from: http://journals.sagepub.com/doi/pdf/10.1177/0734242X07080761 Subscription required to view. 10.1177/0734242X07080761 18229753

[19] Ilemobayo O, Kolade IE Profile of heavy metals from automobile workshops in Akure, Nigeria. J Environ Sci Technol [Internet]. 2008 [cited 2017 Jun 5]; 1 1: 19– 26. Available from: http://scialert.net/fulltext/?doi=jest.2008.19.26

[20] Ipeaiyeda AR, Dawodu M Heavy metals contamination of topsoil and dispersion in the vicinities of reclaimed auto-repair workshops in Iwo Nigeria. Bull Chem Soc Ethiop [Internet]. 2008 [cited 2017 Jun 5]; 22 3: 339– 48. Available from: https://www.ajol.info/index.php/bcse/article/viewFile/61205/49384

[21] Eludoyin OS, Oladele AT Ethno-medicinal studies of trees in University of Port Harcourt, Nigeria. Niger J For. 2013; 43 1&2: 26– 38.

[22] Mmom PC, Fred-Nwagwu FW Analysis of landuse and landcover change around the City of Port Harcourt, Nigeria. Glob Adv Res J Geogr Regional Plan [Internet]. 2013 8 [cited 2017 Jun 5]; 2 5: 76– 86. Available from: http://beta.garj.org/garjgrp/pdf/2013/August/Mmom%20and%20Nwagwu.pdf

[23] Utang BP, Ogba OC, Eludoyin OS. Wind climatology in environmental planning for sustainable air pollution management in the Niger Delta, Nigeria. J Sustain Dev Afr. 2010; 12 1: 369– 83.

[24] Eludoyin OS, Utang PB, Obafemi AA. Geographic Information Systems, urban forestry and climate change: a review. Res J Environ Earth Sci [Internet]. 2012 6 30 [cited 2017 Jun 5]; 4 6: 640– 5. Available from: http://maxwellsci.com/print/rjees/v4-640-645.pdf

[25] Tigist M, Rao VM, Faye G. Determination of essential and non-essential metals concentration in papaya (Carica papaya) seeds, leaves and supporting soil of Odo-Shakiso district in South East Oromia Region, Ethiopia. Int J Res Pharm Chem [Internet]. 2014 [cited 2016 Dec 25]; 4 1: 202– 16. Available from: http://www.ijrpc.com/files/28-454.pdf

[26] Akinola MO, Adenuga AA Determination of the levels of some heavy metals in African pear (dacryodes edulis) marketed in Lagos metropolis, Nigeria. J Appl Sci Environ Manage [Internet]. 2008 3 [cited 2017 Jun 5]; 12 1: 33– 7. Available from: http://www.bioline.org.br/pdf?ja08005

[27] Chukwulobe EE, Saeed MD Assessment of some physicochemical properties and levels of Pb, Cu and Zn in soils of selected dumpsites in Kano Metropolis, North-West, Nigeria. Int J Biol Chem Sci [Internet]. 2014 4 [cited 2016 Dec 24]; 8 2: 717– 26. Available from: https://www.ajol.info/index.php/ijbcs/article/view/107193

[28] Nwaichi EO, Wegwu MO, Nwosu UL. Distribution of selected carcinogenic hydrocarbon and heavy metals in an oil-polluted agriculture zone. Environ Monit Assess [Internet]. 2014 12 [cited 2016 Dec 26]; 186 12: 8697– 706. Available from: https://www.ncbi.nlm.nih.gov/pmc/articles/PMC4210644/ 10.1007/s10661-014-4037-6 PMC4210644 25270365

[29] Khan S, Cao Q, Zheng YM, Huang YZ, Zhu YG. Health risks of heavy metals in contaminated soils and food crops irrigated with wastewater in Beijing, China. Environ Pollut [Internet]. 2008 4 [cited 2016 Dec 24]; 152 3: 686– 92. Available from: http://www.sciencedirect.com/science/article/pii/S0269749107003351 Subscription required to view. 10.1016/j.envpol.2007.06.056 17720286

[30] Nazli MF, Hashim NR. Heavy metal concentrations in an important mangrove species, Sonneratia caseolaris, in Peninsular Malaysia. Environ Asia [Internet]. 2010 [cited 2017 Jun 5]; 3 special issue: 50– 5. Available from: https://www.researchgate.net/publication/285638978_Heavy_Metal_Concentrations_in _an_Important_Mangrove_Species_Sonneratia_caseolaris_in_Peninsular_Malaysia

[31] Tomlinson DC, Wilson JG, Harris CR, Jeffrey DW. Problems in the assessment of heavy metal levels in estuaries and the formation of pollution index. Helgoländer Meeresuntersuchungen [Internet]. 1980 3 [cited 2017 Jun 5]; 33 1: 566– 75. Available from: http://dx.doi.org/10.1007/BF02414780

[32] Ogunmodede OT, Ojo AA, Jegede RO. Evaluation of pollution loads in and around municipal solid waste dumpsite. World Appl Sci J [Internet]. 2016 [cited 2016 Dec 26]; 34 6: 720– 32. Available from: https://www.idosi.org/wasj/wasj34(6)16/7.pdf

[33] USEPA. Soil sampling quality assurance user's guide. EPA/600/8-69/046. Second edition [Internet] 1989 Mar [cited 2017 Jun 11]; 267 p Available from: https://clu-in.org/download/char/soilsamp.pdf

[34] Patil GP. Composite sampling. In El-Shaarawi AH, Piegorsch WW( eds). Encyclopedia of Environmetrics, [Internet]. 2002 [cited 2017 Jun 11]; 1: 387– 391 Available from: http://sites.stat.psu.edu/~gpp/pdfs/TR2001-0202.pdf

[35] Chary NS, Kamala CT, Raj DS. Assessing risk of heavy metals from consuming food grown on sewage irrigated soils and food chain transfer. Ecotoxicol Environ Saf [Internet]. 2008 3 [cited 2017 Jun 5]; 69 3: 513– 24. Available from: http://www.sciencedirect.com/science/article/pii/S0147651307000851 Subscription required to view. 10.1016/j.ecoenv.2007.04.013 17555815

[36] Ogunkunle AT, Bello OS, Ojofeitimi OS. Determination of heavy metal contamination of street-vended fruits and vegetables in Lagos State, Nigeria. Int Food Res J [Internet]. 2014 [cited 2017 Jun 5]; 21 5: 1725– 30. Available from: http://www.ifrj.upm.edu.my/21%20(05)%202014/3%20IFRJ%2021%20(05)%202014%20Ogunkunle%20619.pdf

[37] Uwah EI, Gimba MS, Gwaski PA. Determination of Zn, Mn, Fe and Cu in spinach and lettuce cultivated in Potiskum, Yobe State, Nigeria. J Agric Econ Dev [Internet]. 2012 10 [cited 2017 Jun 5]; 1 4: 69– 74. Available from: http://academeresearchjournals.org/download.php?id=775724227341794624.pdf

[38] Bigdeli M, Seilsepour M Investigation of metals accumulation in some vegetables irrigated with waste water in Shahre Rey-Iran and toxicological implications. Am-Eurasian J Agric Environ Sci. 2008; 4 1: 86– 92.

[39] Hailemariam T, Aregahegn A, Bekele T, Madhusudhan A. Investigation of the levels of selected metals in edible and medicinal fruits grown in Dilla, Ethiopia. Res J Chem Environ Sci. [Internet]. 2015 8 [cited 2017 Jun 5]; 3 4: 44– 53 Available from: http://www.aelsindia.com/rjcesaugust2015/7f.pdf

[40] Suplemental guidance for developing soil screening levels for superfund sites [Internet]. Washington, D.C.: U.S. Environmental Protection Agency; 2002 12 [cited 2016 Dec 26] 187 p. Available from: https://nepis.epa.gov/Exe/ZyPURL.cgi?Dockey=91003IJK.TXT

[41] Environmental guidelines and standards for the petroleum industry in Nigeria (EGASPIN) [Internet]. Lagos, Nigeria: Department of Petroleum Resources: 1991 [revised 2002; cited 2017 Jun 5] 415 p. Available from: https://www.scribd.com/doc/233481740/The-Environmental-Guidelines-and-Standards-for-the-Petroleum-Industry-in-Nigeria-EGASPIN-2002 Subscription required to view.

[42] Chiroma TM, Ebewele RO, Hymore FK. Comparative assessment of heavy metal levels in soil, vegetables and urban grey waste water used for irrigation in Yola and Kano. Int Refereed J Eng Sci [Internet]. 2014 2 [cited on 2016 Dec 26]; 3 2: 1– 9. Available from: http://www.irjes.com/Papers/vol3-issue2/A03020109.pdf

[43] Lacatusu R. Appraising levels of soil contamination and pollution with heavy metals. In Heineke HJ, Eckelmann W, Thomasson AJ, Jones RJA, Montanarella L, Buckey B ( eds). European Soil Bureau Research Report, Office for Officials Publications of the European Communities Luxembourg [Internet]. 1998 [Updated 2011; cited 2017 Jun 11]; 4: 393– 402 Available from http://eusoils.jrc.ec.europa.eu/ESDB_Archive/eusoils_docs/doc_ESBN.html

[44] Abdallah SA, Uzairu A, Kagbu JA, Okunola OJ. Assessment of heavy metals bioaccumulation by Eleusine indica from refuse dumpsites in Kaduna Metropolis, Nigeria. J Environ Chem Ecotoxicol [Internet]. 2012 5 22 [cited 2017 Jun 5]; 4 9: 153– 60. Available from: http://www.academicjournals.org/journal/JECE/article-abstract/397DBF63259

[45] Obasi NA, Akubugwo E, Kalu KM, Ugbogu AE, Okorie UC. Toxicological assessment of various metals on selected edible leafy plants of Umuka and Ubahu dumpsites in Okigwe of Imo State, Nigeria. J Exp Biol Agric Sci [Internet]. 2013 12 [cited 2016 Dec 25]; 1 6: 441– 53. Available from: http://www.jebas.org/wp-content/uploads/2014/09/Obasi-et-al-JEBAS.pdf

[46] Ololade IA. An assessment of heavy-metal contamination in soils within auto-mechanic workshops using enrichment and contamination factors with geoaccumulation indexes. J Environ Prot [Internet]. 2014 8 [cited 2017 Jun 6]; 5: 970– 82. Available from: http://dx.doi.org/10.4236/jep.2014.511098

[47] Odoh R, Agbaji EB, Kagbu JA. Assessment of trace metals pollution in auto-mechanic workshop in some selected local government area of Benue State, Nigeria. Int J Chem [Internet]. 2011 12 [cited 2016 Dec 26]; 3 4: 78– 88. Available from: http://dx.doi.org/10.5539/ijc.v3n4p78

[48] Orisakwe OE, Asomugha R, Afonne OJ, Anisi CN, Obi E, Dioka CE. Impact of effluents from a car battery manufacturing plant in Nigeria on water, soil, and food qualities. Arch Environ Health [Internet]. 2004 1 [cited 2017 Jun 6]; 59 1: 31– 6. Available from: http://www.tandfonline.com/doi/abs/10.3200/AEOH.59.1.31-36?needAccess=true&journalCode=vzeh20 Subscription required to view. 10.3200/AEOH.59.1.31-36 16053207

[49] Hanlon EA. Jr Soil pH and electrical conductivity: a county extension soil laboratory manual [Internet]. Gainesville, FL: The Institute of Food and Agricultural Sciences Extension, University of Florida; 1993 4 [reviewed 2015 Aug; cited 2016 Dec 26] 10 p. Available from: http://edis.ifas.ufl.edu/ss118

[50] Mohd-Aizat A, Mohamad-Roslan MK, Sulaiman WN, Karam DS. The relationship between soil pH and selected soil properties in 48 years logged-over forest. Int J Environ Sci [Internet]. 2014 [cited 2016 Dec 26]; 4 6: 1129– 40 Available from: http://www.ipublishing.co.in/ijesarticles/fourteen/articles/volfour/EIJES41108.pdf

[51] Oyedele DJ, Obioh IB, Adejumo JA, Oluwole AF, Aina PO, Asubiojo OI. Lead contamination of soils and vegetation in the vicinity of a lead smelter in Nigeria. Sci Total Environ [Internet]. 1995 11 30 [cited 2017 Jun 6]; 172 2–3: 189– 95. Available from: http://www.sciencedirect.com/science/article/pii/0048969795048103 Subscription required to view.

[52] Yoon J, Cao X, Zhou Q, Ma LQ. Accumulation of Pb, Cu and Zn in native plants growing on a contaminated Florida site. Sci Total Environ [Internet]. 2006 9 15 [cited 2017 Jun 6]; 368 2–3: 456– 64. Available from: http://www.sciencedirect.com/science/article/pii/S0048969706000945 Subscription required to view. 10.1016/j.scitotenv.2006.01.016 16600337

[53] Rice KV, Walker EM Jr, Wu M, Gillete C, Blough ER. Environmental mercury and its toxic effects. J Prev Med Public Health [Internet]. 2014 3 [cited 2017 Jun 6]; 47 2: 74– 83. Available from: https://www.ncbi.nlm.nih.gov/pmc/articles/PMC3988285/ 10.3961/jpmph.2014.47.2.74 PMC3988285 24744824

[54] Beesley L, Moreno-Jimenez E, Clemente R, Lepp N, Dickinson N. Mobility of arsenic, cadmium and zinc in a multi-element contaminated soil profile assessed by in-situ soil pore water sampling, column leaching and sequential extraction. Environ Pollut [Internet]. 2010 1 [cited 2017 Jun 6]; 158 1: 155– 60. Available from: http://www.sciencedirect.com/science/article/pii/S0269749109003595 Subscription required to view. 10.1016/j.envpol.2009.07.021 19683374

[55] Clemente R, Dickinson NM, Lepp NW. Mobility of metals and metalloids in a multi-element contaminated soil 20 years after cessation of the pollution source activity. Environ Pollut [Internet]. 2008 9 [cited 2017 Jun 6]; 155 2: 254– 61. Available from: http://www.sciencedirect.com/science/article/pii/S0269749107005775 Subscription required to view. 10.1016/j.envpol.2007.11.024 18249071

[56] Chunilall V, Kindness A, Jonnalagadda SB. Heavy metal uptake by two edible Amaranthus herbs grown on soils contaminated with lead, mercury, cadmium, and nickel. J Environ Sci Health B [Internet]. 2005 [cited 2017 Jun 6]; 40 2: 375– 84. Available from: http://www.tandfonline.com/doi/abs/10.1081/PFC-200045573?journalCode=lesb20 Subscription required to view. 10.1081/PFC-200045573 15825688

[57] Krawczyk J, Klink A, Letachowicz B, Wisocka M. A study of heavy metal content in mouse-ear hawkweed, Hieracium pilosella L. and the soil from its habitats within the Wielun area. Pol J Environ Stud. 2006; 15 2A(I: 116– 20.

[58] Sekara A, Poniedzialek M, Ciura J, Jedrszcyk E. Zinc and copper accumulation and distribution in tissues of nine crops: implication for phytoremediation. Pol J Environ Stud [Internet]. 2005 [cited 2017 Jun 6]; 14 6: 829– 35. Available from: http://www.pjoes.com/pdf/14.6/829-835.pdf

[59] John R, Ahmad P, Gadgil K, Sharma S. Heavy metal toxicity: effect on plant growth, biochemical parameters and metal accumulation by Brassica juncea L. Int J Plant Prod [Internet]. 7 2009 [cited 2016 Dec 26]; 3 3: 65– 76. Available from: http://ijpp.gau.ac.ir/article_653_06af184ee210bcaafe0d7cbcc16398fc.pdf

[60] Cortez LA, Ching JA Heavy metal concentration of dumpsite soil and accumulation in Zea mays (corn) growing in a closed dumpsite in Manila, Philippines. Int J Environ Sci Dev [Internet]. 2014 2 [cited 2016 Dec 26]; 5 1: 77– 80. Available from: http://www.ijesd.org/papers/454-P10003.pdf

[61] Kalagbor IA, Naifa PB, Umeh JN. Analysis of heavy metals in four fruits from Sii and Zaakpon Communities in Khana, Rivers State. Int J Emerg Technol Adv Eng [Internet]. 2014 5 [cited 2017 Jun 6]; 4 5: 827– 31. Available from: http://www.ijetae.com/files/Volume4Issue5/IJETAE_0514_126.pdf

[62] Chen TB, Zheng YM, Lei M, Huang ZC, Wu HT, Chen H, Fan KK, Yu K, Wu X, Tian QZ. Assessment of heavy metal pollution in surface soils of urban parks in Beijing, China. Chemosphere [Internet]. 2005 7 [cited 2016 Dec 26]; 60 4: 542– 51. Available from: http://www.sciencedirect.com/science/article/pii/S0045653505000846 Subscription required to view. 10.1016/j.chemosphere.2004.12.072 15950046

[63] Blake L, Goulding KW Effects of atmospheric deposition, soil pH and acidification on heavy metal contents in soils and vegetation of semi-natural ecosystems at Rothamsted Experimental Station, UK. Plant Soil [Internet]. 2002 3 [cited 2017 Jun 6]; 240 2: 235– 51. Available from: https://link.springer.com/article/10.1023/A:1015731530498 Subscription required to view.

[64] Tangahu BV, Abdullah SR, Basri H, Idris M, Anuar N, Mukhlisin M. A review on heavy metals (As, Pb, and Hg) uptake by plants through phytoremediation. Int J Chem Eng [Internet]. 2011 [cited 2017 Jun 6]; 2011 2011: 1– 31. Available from: https://www.hindawi.com/journals/ijce/2011/939161/

[65] Zauro SA, Dabai MU, Tsafe AI, Umar KJ, Lawal AM. Extent of some heavy metals contamination in soil of farmlands around Sokoto Metropolis. Eur Sci J [Internet]. 2013 1 [cited 2017 Jun 6]; 9 3: 30– 6. Available from: http://eujournal.org/index.php/esj/article/viewFile/729/778

[66] Sharma P, Dubey RS Lead toxicity in plants. Braz J Plant Physiol [Internet]. 2005 [cited 2017 Jun 6]; 17 1: 35– 52. Available from: http://scielo.br/pdf/bjpp/v17n1/a04v17n1.pdf

[67] Ang HH, Lee EL, Matsumato K. Analysis of lead content in herbal preparation in Malaysia. Hum Exp Toxicol [Internet]. 2003 8 1 [cited 2017 Jun 6]; 22 8: 445– 51. Available from: http://journals.sagepub.com/doi/abs/10.1191/0960327103ht382oa Subscription required to view. 10.1191/0960327103ht382oa 12948085

[68] Wells EC, Terry RE, Parnell JJ, Hardin PJ, Jackson MW, Houston SD. Chemical analyses of ancient anthrosols in residential areas at Piedras Negras, Guatemala. J Archaeol Sci [Internet]. 2000 5 [cited 2017 Jun 6]; 27 5: 449– 62. Available from: http://www.sciencedirect.com/science/article/pii/S0305440399904909 Subscription required to view.

[69] Olayiwola OA. Accumulation and contamination of heavy metals in soil and vegetation from industrial area of Ikirun, Osun State, Nigeria. Glob J Pure Appl Chem Res [Internet]. 2013 6 [cited 2017 Jun 6]; 1 1: 25– 34. Available from: http://www.eajournals.org/journals/global-journal-of-pure-and-applied-chemistry-research-gjpacr/ vol-1-issue-1-june-2013/accumulation-and-contamination-of-heavy-metals-in-soil-and-vegetation-from-industrial-area-of-ikirun-osun-state-nigeria/

[70] Agbaji EB, Abechi SE, Emmanuel SA. Assessment of heavy metals level of soil in Kakuri industrial area of Kaduna, Nigeria. J Sci Res Rep [Internet]. 2015 [cited 2017 Jun 6]; 4 1: 68– 78. Available from: http://www.sciencedomain.org/abstract/6328

